# The Study of DNA Methyltransferase-3B Promoter Variant Genotype among Iranian Sporadic Breast Cancer Patients

**Published:** 2014-05

**Authors:** Ebrahim Eftekhar, Mozhgan Rasti, Fakhraddin Nahgibalhossaini, Yasaman Sadeghi

**Affiliations:** 1 Deparment of Biochemistry, School of Medicine, Shiraz University of Medical Sciences, Shiraz, Iran;; 2 Student Research Committee, School of Medicine, Shiraz University of Medical Sciences, Shiraz, Iran;; 3 Department of Biochemistry, School of Medicine, Hormozgan University of Medical Sciences, Bandar Abbas, Iran

**Keywords:** DNAmethyltransferase-3B, Polymorphism, Breast cancer

## Abstract

**Background: **DNA methyltransferase-3B (DNMT3B) is an important enzyme responsible for maintaining the DNA methylation pattern in eukaryotic cells. In this study we have investigated the correlation between the 46359C→T polymorphism in the DNMT3B gene and the risk of breast cancer incidence among sporadic breast cancer patients in Fars Province, Southern Iran.

**Methods: **In this case-control study, 100 breast cancer patients and 138 healthy control subjects were genotyped for the DNMT3B gene by the polymerase chain reaction-restriction fragment length polymorphism method.

**Results:** The genotype frequency in the case (CC 27%, CT 47%, TT 26%) group significantly (P=0.008) differed from the control (CC 19.56%, CT 67.3%, TT 13%) group. We observed a decreased association between the CT genotype and lymph node involvement in breast cancer patients. Our results have shown that in comparison to the homozygous CC genotype carriers the DNMT3B-CT genotype has a significantly lower risk for breast cancer (OR=0.515, 95% CI=0.267-0.994, P=0.048).

**Conclusion: **Our case-control study showed that the CT genotype was significantly associated with decreased breast cancer risk. Consistent with these results, a significant decrease of CT genotype among lymph node positive breast cancer patients was observed. However, a larger study population with more clinical data is needed to confirm these results.

## Introduction

Breast cancer is the most frequently diagnosed cancer and the leading cause of cancer death among women.^1^ It has been reported that breast cancer affects women in Iran at least one decade earlier than in developed countries.^2^ The molecular mechanisms that contribute to the development and progression of breast cancer are poorly understood. During the past decade it became evident that epigenetic alteration plays an important role in neoplastic transformation.^3^^-^^5^

DNA methylation is a major epigenetic mechanism that has an important role in chromosomal stability and gene expression in mammalian cells.^6^^-^^8^ Aberrant promoter methylation of tumor suppressor genes is closely related with loss of their function.^9^^,^^10^ DNA methyltransferases, of which three active forms have been identified (DNMT1, DNMT3A and DNMT3B) catalyze DNA methylation. DNMT1 maintains the levels and patterns of methylated DNA during mitosis, whereas DNMT3A and DNMT3B are primarily responsible for de novo methylation.^3^^,^^11^^,^^12^ De novo hypermethylation of promoter CpG islands has been identified as a possible mechanism for tumor suppressor gene inactivation in human cancer cells.^13^^,^^14^ DNMT3B plays an important role in tumorigenesis, and overexpression of DNMT3B has been reported in tumors. However DNMT1 and DNMT3A have been found to be only modestly overexpressed at lower frequencies.^15^^,^^16^ Up regulation of DNMT3B is dramatically associated with a higher histopathological grade of breast tumors as well as proliferation of marker Ki67 and negative estrogen receptor-α expression – all indicative of possible DNMT3B involvement in breast tumor progression and metastasis.^17^

The DNMT3B gene, located on chromosome 20q11.2, contains a C to T transition polymorphism (C46359T, GenBank accession no. AL035071) in the promoter region of the DNMT3B gene, -149 base pairs from the transcription start site.^18^ Many reports have shown that the DNMT3b C/T polymorphism may change the enzyme methylating activity and thereby influence the incidence of cancer susceptibility.^18^^-^^20^ However, there is no consensus in the literature regarding an association between DNMT3B genotypes and the risk of different cancers.^18^^-^^20^ To the best of our knowledge, the association between DNMT3B polymorphism and breast cancer risk has been reported in a single British study.**^19^** To date, this has not been explored in Iranian populations.

Therefore, we investigated the association between DNMT3B genotype and the risk of breast cancer incidence among sporadic breast cancer patients in Fars Province, Southern Iran.

## Materials and Methods

*Study Subjects*

A total of 100 sporadic breast tumor samples (95 fresh and 5 paraffin-embedded) were obtained from the Department of Pathology, Shiraz University of Medical Sciences, Shiraz, Iran from 2003 to 2006. Fresh samples were snap-frozen immediately after surgery and stored at -70°C. All samples were subjected to re-evaluation of the original histological diagnosis by an expert pathologist who also selected representative areas of the tissue sections for DNA extraction and further molecular analysis. Each patient’s clinicopathological information that included age, tumor size, type, grade and site, estrogen and progesterone receptor and lymph node involvement status was obtained from hospital records. The 138 healthy control females, matched for age with the case subjects, were selected from a pool of cancer-free subjects who volunteered to join the epidemiology survey during the same period. For the control group, normal genomic DNA was prepared from blood lymphocytes. This investigation was approved by the Ethics Committee of Shiraz University of Medical Sciences. 

*DNA Extraction and DNMT3B Genotyping*

**Figure 1:**
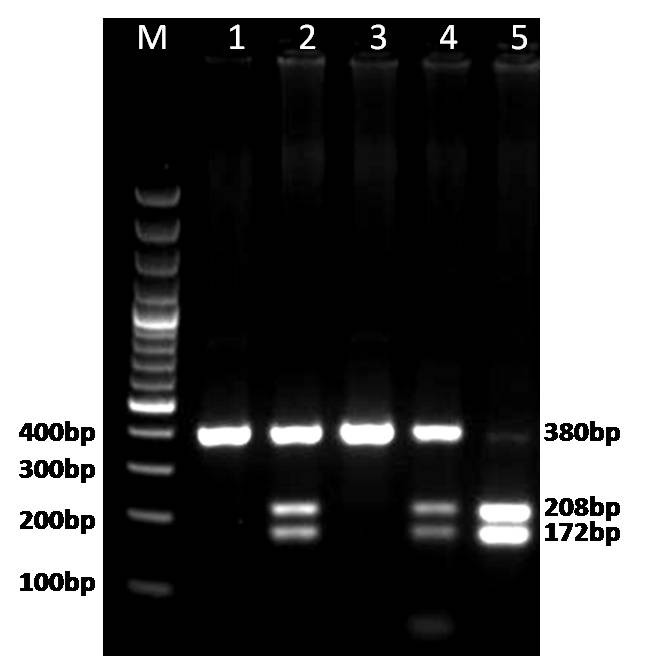
PCR-RFLP based genotyping of DNMT3B C46359T. Lanes 1 and 3: CC wild type. Lanes 2 and 4: CT heterozygotes. Lane 5: TT homozygote variant.

**Figure 2:**
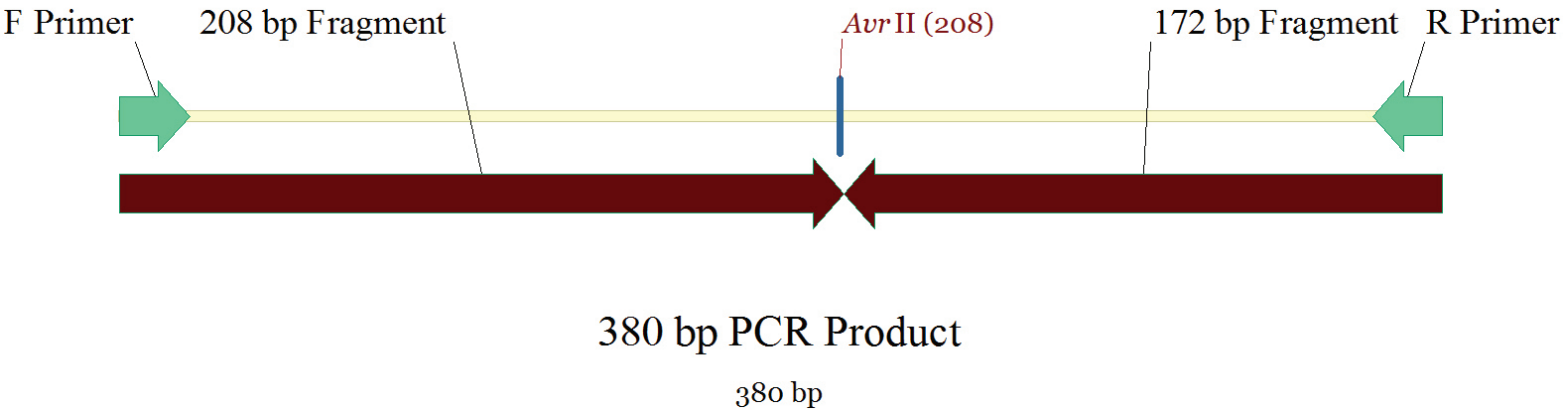
Genetic map of DNMT3B with primers’ binding sites and the AvrII restriction site by using Vector NTI 10.0 software.

Genomic DNA was isolated from tumor samples (case group) and peripheral blood lymphocytes (control group) using a Cinnagen genomic DNA purification kit (Cinnagen, Iran). The purity and concentration of DNA were assessed by spectrophotometric measurement of absorbance at 260 and 280 nm. DNMT3B C/T polymorphism was analyzed by polymerase chain reaction-restriction fragment length polymorphism (PCR-RFLP). The PCR sense (5´-TGCTGTGACAGGCAGAGCAG-3´) and antisense (5´-GGTAGCCGGGAACTCCACGG-3´) primers were used to amplify the target DNA as previously described.^18^ Briefly, we used 25 μl of PCR mixture that contained 100-300 ng of DNA template, 12.5 pmol of each primer (Takapoo Zist Company, Iran), 0.1 mmol/L of each deoxynucleotide triphosphate, 1×PCR buffer (50 mmol/L KCl, 10 mmol/L tris-HCl, and 0.1% Triton X-100), 2.0 mmol/L MgCl_2_, and 1.25 U Taq polymerase (Cinnagen Company, Iran). The PCR amplification profile consisted of an initial denaturation step at 95°C for 5 min, 35 cycles of denaturation at 95°C for 30 s, annealing at 65°C for 30 s, and extension at 72°C for 30 s. This was followed by a further extension step at 72°C for 10 min. The 380 bp PCR products were digested overnight with 5 units of *Avr*II (Vivantis Company, Malaysia) at 37°C and separated on 2% agarose gels. The digested product was visualized by red gel staining under UV illumination. The variant T allele has a *Avr*II restriction site that results in two bands (208 bp and 172 bp) whereas the wild-type C allele lacks the *Avr*II restriction site, thus producing a single 380-bp band. Therefore the heterozygote alleles were expected to have three bands (380, 208, and 172 bp; figure 1). We used VECTOR NTI 10.0 software (IBI, USA) to draw the genetic map for DNMT3B with primers' binding sites and the *Avr*II restriction site (figure 2).

*Statistical Analysis*

The difference in frequency distributions of the DNMT3B genotypes and allelotypes between the patients and the control group were analysed using the chi-square test. The odds ratios (ORs) and 95% confidence intervals (CIs) for the DNMT3B genotype were calculated by logistic regression analysis, with adjustment for age. A P value *<*0.05 was considered statistically significant. All data were analyzed using SPSS 12.0 software.

## Results

The clinicopathological characteristics of the study subjects are shown in table 1. The mean±SD age was 48.51±15.32 (range: 16-70 years) for the case patients and 47.41±17.52 years (range: 18-78 years) for the control subjects. A total of 87.8% of breast cancer patients were classified as invasive ductal carcinoma, 9.8% as invasive lobular carcinoma, and 2.4% had other less common carcinomas that included medullary, papillary and tubular carcinomas. No significant differences were found in the mean age or sex distribution, which suggested that the cases and control were adequately matched. The frequency of DNMT3B 46359 C→T polymorphism in cancer cases and control is summarized in table 2. There were no significant differences between frequency of alleles in the case and control groups (table 2). However, the frequency of T allele was 6% higher in case patients (0.5) compared to the control group (0.47). The genotype frequency in the case group (CC 27%, CT 47%, TT 26%) was significantly (P=0.008) different from the control group (CC 19.56%, CT 67.3%, TT 13%). When the CC genotype was used as the reference group the TT genotype was not associated with risk (OR=1.3, 95% CI=0.56-2.99, P=0.27). However there was a significant association with the CT genotype and decreased risk for breast cancer (OR=0.51, 95% CI=0.26-0.99, P=0.04). In addition, the combined variant genotypes (CT+TT) had no significant decrease in risk of breast cancer (OR=0.601, 95% CI=0.3-12.195, P=0.14). The associations between the DNAMT3B genotype and breast cancer stratified according to age, grade, tumor size, lymph node involvement and histopathological type in case patients are shown in table 3. When adjusted by age, a significant association between size, grade, side and type of tumor, estrogen or progesterone status and DNMT3B genotype was not observed (table 3). However, there was a significant decrease (P=0.02) in association of CT genotype with lymph node involvement in patients (OR=0.37, 95% CI=0.16-0.86). We found that the CT genotype only occurred in 37% of malignant tumors that had positive lymph nodes.

**Table 1:** Clinicopathological characteristics of study subjects

**Variables**	**Breast cancer ** **n (%)**	**Control ** **n (%) **
Sex		
Female	100	138
Age (years)		
<50	60 (60)	92 (66.7)
≥50	40 (40)	46 (33.33)
Grade		
I	13 (15.7)	
П and III	70 (84.3)	
Tumor type		
Invasive ductal carcinoma	72 (87.8)	
Invasive lobular carcinoma	8 (9.8)	
Other	2 (2.4)	
Tumor size		
≤2 mm	30 (34.1)	
<2 - <5 mm	46 (52.3)	
≥5 mm	12 (13.6)	
Tumor site		
Right	39 (53.4)	
Left	34 (46.6)	
Lymph node involvement		
Positive	44 (51.8)	
Negative	41 (48.2)	
Estrogen receptor		
Positive	59 (72.8)	
Negative	22 (27.2)	
Progesterone receptor		
Positive	49 (60.5)	
Negative	32 (39.5)	

**Table 2:** The frequency of DNMT3B 46359 C→T polymorphism in cancer cases and controls

**Genotype**	**Breast cancer**		**Control**		** Adjusted odds ratio^a^** **(95% CI, P value)**
	**n**	**%**	**n**	**%**	
Genotype frequency					
CC	27	27	27	19.56	1
CT	47	47	93	67.3	0.51 (0.26-0.99, 0.04)
TT	26	26	18	13	1.30 (0.56-2.99, 0.27)
CT+TT	73	73	111	80.3	0.60 (0.30-1.19, 0.14)
Allele frequency					
C	50	50	73	53	P<0.05
T	50	50	65	47	

^a^Adjusted for age.

**Table 3:** Stratification analysis of DNMT3B genotype frequencies

**Adjusted OR (95% CI, P value)**				
**Variables**	**CC (ref)**	**CT**	**TT**	**CT+TT**
Age (years)				
<50	1	0.62 (0.26-1.47, 0.28)	1.2 (0.37-2.33,0.54)	0.78 (0.64-1.33, 0.21)
≥50	1	0.63 (0.18-2.13, 0.18)	1.12 (0.46-2.02,0.25)	0.83 (0.32-1.89, 0.17)
Grade				
I	1	0.62 (0.28-1.29, 0.62)	1.31 (0.5-3.43, 0.57)	0.72 (0.34-1.51, 0.38)
П and III	1	0.33 (0.09-1.19, 0.09)	0.28 (0.03-2.6, 0.26)	0.32 (0.09-1.11, 074)
Tumor size				
<2 mm	1	0.69 (0.24-1.19, 0.69)	2.11 (0.63-7.06, 0.22)	0.93 (0.34-2.53, 0.89)
<2 - <5 mm	1	0.44 (0.20-0.98, 0.09)	0.65 (0.2-1.96, 0.45)	0.48 (0.2-1.03, 0.06)
≥5 mm	1	0.69 (0.12-3.83, 0.67)	3.52 (0.6-2.67, 0.32)	1.16 (0.2-5.33, 0.85)
Lymph node involvement				
Yes	1	0.37 (0.16-0.86, 0.02)	1.11 (0.4-3.03, 0.84)	0.49 (0.2-1.08, 0.07)
No	1	0.65 (0.2-1.7, 0.37)	1.58 (0.5-4.9, 0.4)	0.80 (0.3-1.9, 0.6)
Histology				
Ductal carcinoma	1	0.57 (0.2-1.2, 0.13)	0.33 (0.5-3.3, 0.5)	0.69 (0.3-1.4, 0.3)
Lobular carcinoma	1	1.11 (0.1-1.46, 0.9)	2.31 (0.4-3.4, 0.2)	1.6 (0.19-5.4, 0.6)

## Discussion

The mechanism of the association between DNMT3B 149 C→T polymorphism and the risk of cancer is not clearly understood. According to the underlying hypothesis, the C→T transition may up regulate DNMT3B expression, resulting in increased susceptibility toward aberrant de novo methylation of CpG islands of the promoter in some tumor suppressor genes and thereby increase cancer risk.^15^^,^^21^ In agreement with this hypothesis, Shen et al. have reported that carriers of T alleles, particularly heterozygous (CT), had a significant increase in lung cancer risk compared to the homozygous CC genotype.^18^ However, we found that the CT genotype was significantly associated with decreased risk (2 fold) of breast cancer (OR=0.51, 95% CI=0.26-0.99, P=0.04). Since we were unable to adjust for environmental risk factors (i.e., alcohol, smoking) we could not exclude the possibility that such confounding factors might have led to a type I error. Possibly both factors were involved, therefore this discrepancy could be due to different functions of DNMT3B in different cell types. It has been reported that several spliced forms of DNMT3B, with different enzyme activity are expressed in a tissue specific manner.^15^^,^^22^^,^^23^ We also observed a decreased association between the CT genotype and lymph node involvement in breast cancer patients, which suggested that genetic susceptibility might play an important role in metastatic properties of aggressive breast cancer tumors. The results of other investigations regarding the association between DNMT3B single nucleotide polymorphism (SNPs) and the risk of cancer were conflicting. Wu et al. demonstrated that the C/T polymorphism was not associated with up regulation of DNMT3B and increased risk of hepatocellular carcinoma.^23^ These researchers observed a similar pattern of DNMT3B genotype among hepatocellular carcinoma patients (n=100) and healthy subjects (n=140).^23^ An investigation in north China showed that the C/T polymorphism was not associated with susceptibility to gastric cardiac adenocarcinoma.^24^ Montgomery et al. genotyped 352 cases and 258 controls from a British population and found that carriers of C alleles showed significant increases in breast cancer risk.^19^ Their findings did not agree with the hypothesis in which the carrier of T alleles should have higher susceptibility to cancer. They suggested this inconsistency might be an artifact that resulted from a chance variation or it might point to differing influences of promoter methylation in this type of cancer. In contrast to the research of Montgomery et al., the results of two other studies showed that carriers of T alleles, particularly the TT genotype, notably increased the risks of squamous cell carcinoma of the head and neck (SCCHN) and prostate cancer.^25^^,^^26^ The findings of the current study were not consistent with the previous conflicting data. However, the current study’s limitation was the inability to adjust the study data for confounding factors. As the results of different studies have shown, there is no consensus regarding the association between DNMT3B genotypes and the risk of cancer. These inconsistent results may be due to factors such as small sample size, different ethnic groups, geographic areas and inadequate adjustment for confounding factors.

## Conclusion

Our case-control study showed that the CT genotype was significantly associated with decreased risk of breast cancer in our studied groups. Consistent with these results, we observed a significant decrease in CT genotype among lymph node positive breast cancer patients. Further studies with larger samples size and more clinical data are required to confirm these results.
